# Supplementary material for: Pattern and correlates of physical activity and sedentary behaviours of pregnant women in Ibadan, Nigeria: Findings from Ibadan pregnancy cohort study
Source: PLOS Glob Public Health. 2022 Oct 12;2(10):e0001153. doi: 10.1371/journal.pgph.0001153 (PMC10021993; doi:10.1371/journal.pgph.0001153)
Supplement: Attachment — Submitted filename: Response to the Editor_PGPH_R1_Physical activity.docx [file pgph.0001153.s001.docx]

Response to the Editor: Plos Global Public Health

Journal Requirements:

1. Please amend your detailed Financial Disclosure statement.

This is published with the article. It must therefore be completed in full sentences and contain the exact wording you wish to be published.

a. State the initials, alongside each funding source, of each author to receive each grant.

– Not Applicable

b. State what role the funders took in the study. If the funders had no role in your study, please state: “The funders had no role in study design, data collection and analysis, decision to publish, or preparation of the manuscript.”

- Included

c. If any authors received a salary from any of your funders, please state which authors and which funders.

– Not Applicable

- This manuscript is one of the outputs of my PhD research that was supported by CARTA - the Consortium of Advanced Research Training in Africa. It was CARTA that received the grant from the funders not the author hence the acknowledgement of funding is presented as follows.
  - *"This research was supported by the Consortium for Advanced Research Training in Africa (CARTA). CARTA is jointly led by the African Population and Health Research Center and the University of the Witwatersrand and funded by the Carnegie Corporation of New York (Grant No. G-19-57145), Sida (Grant No:****54100113****), Uppsala Monitoring Center, Norwegian Agency for Development Cooperation (Norad), and by the Wellcome Trust [reference no. 107768/Z/15/Z] and the UK Foreign, Commonwealth & Development Office, with support from the Developing Excellence in Leadership, Training and Science in Africa (DELTAS Africa) programme. The statements made and views expressed are solely the responsibility of the Fellow.* *Ikeola Adeoye is a CARTA PhD fellow. For the purpose of open access, the author has applied a CC BY public copyright licence to any Author Accepted Manuscript version arising from this submission." The funders had no role in study design, data collection and analysis, decision to publish, or preparation of the manuscript*

2. In the online submission form, you indicated that "The Ibadan Pregnancy Cohort Study is an ongoing study it is premature to put the data in the public domain now. Besides the datasets generated contain potentially identifying and confidential information. However, data sharing could be considered at a later time on reasonable request from the corresponding author if they meet the criteria for accessing confidential data.".

- Rephrased

***(Availability of data and materials:*** *The datasets generated and/or analysed during the current study are not publicly available because they contain potentially identifying and confidential information but are available from the UI/UCH Ethics Committee (uiuchec@gmail.com) on reasonable request if it meets the criteria for accessing confidential data.)*

3. All PLOS journals now require all data underlying the findings described in their manuscript to be freely available to other researchers, either 1. In a public repository, 2. Within the manuscript itself, or 3. Uploaded as supplementary information. This policy applies to all data except where public deposition would breach compliance with the protocol approved by your research ethics board. If your data cannot be made publicly available for ethical or legal reasons (e.g., public availability View Letter https://www.editorialmanager.com/pgph/ViewLetter.aspx?id=202173&l... 1 of 3 7/13/2022, 11:02 PM would compromise patient privacy), please explain your reasons by return email and your exemption request will be escalated to the editor for approval. Your exemption request will be handled independently and will not hold up the peer review process, but will need to be resolved should your manuscript be accepted for publication. One of the Editorial team will then be in touch if there are any issues.

- AN APPLICATION FOR EXEMPTION IS SENT BY MAIL

4. Please review your reference list to ensure that it is complete and correct. If you have cited papers that have been retracted, please include the rationale for doing so in the manuscript text, or remove these references and replace them with relevant current references. Any changes to the reference list should be mentioned in the rebuttal letter that accompanies your revised manuscript. If you need to cite a retracted article, indicate the article’s retracted status in the References list and also include a citation and full reference for the retraction notice
   - NO RETRACTED ARTICLE CITED

Sincerely,

Thank you

Ikeola Adeoye
